# Development of self-care strategies to promote young Iranian women’s sexual health: an explanatory sequential mixed method study protocol

**DOI:** 10.1186/s12978-023-01692-y

**Published:** 2023-10-05

**Authors:** Batool Bonyadpour, Raziyeh Maasoumi, Maryam Nekoolaltak

**Affiliations:** 1 Department of Midwifery and Reproductive Health, School of Nursing and Midwifery, Tehran University of Medical Sciences, P.O. Box: 1419733171, Tehran, Iran; 2 Department of Reproductive Health, Nursing and Midwifery Care Research Centre, School of Nursing and Midwifery, Tehran University of Medical Sciences, P.O. Box: 1419733171, Tehran, Iran

**Keywords:** Self-care, Sexual health, Single young women

## Abstract

**Background:**

In contemporary Iran, the nation's traditional and deeply religious society is currently experiencing swift transformations in its moral, cultural, and social aspects. It is, therefore, not surprising to observe shifts in people's attitudes toward sexuality, largely attributed to the profound impact of widespread social networks, the proliferation of information technology, and increased levels of education. Unmarried young women may potentially face adverse consequences from engaging in extramarital sexual relationships across various aspects of their lives. Acknowledging the pivotal role of self-care in influencing the sexual behaviors of young women, the objective of this study is to compile a comprehensive list of self-care strategies aimed at improving the sexual well-being of young, single Iranian women.

**Methods:**

The research will unfold in three distinct phases: Phase 1: Explanatory Sequential Mixed-Method Study This initial phase encompasses both quantitative and qualitative aspects. It begins with a cross-sectional survey, where we will gather data from 400 unmarried female students aged 18 to 29 years, utilizing a cluster random sampling method at Kerman University of Medical Sciences. Data collection will involve the use of a researcher-designed questionnaire. Subsequently, the qualitative phase will involve conducting in-depth, semi-structured interviews with female students from the University. To analyze this qualitative data, we will employ the content analysis approach. The findings obtained from both phases will be combined. Phase 2: Narrative Review In the second stage of the study, we will conduct an extensive narrative review to explore existing strategies related to the subject matter comprehensively. This review will serve as the foundational basis for our subsequent analysis. Phase 3: Strategy Prioritization In the final phase, we will prioritize the proposed strategies using a nominal group process, soliciting expert advice. This step will result in the definitive list of strategies that emerge from the study.

**Discussion:**

This study pioneers the field of sexual health, with the goal of developing a protocol for creating self-care strategies based on the perspectives of young, unmarried Iranian women. It offers potential evidence-based insights into current developments in the physical, psychological, and social aspects of sexual health within this demographic. Additionally, it aims to furnish essential information to healthcare policymakers regarding the sexual health of young women.

## Plain language summary

The convergence of the Iranian cultural landscape with the widespread presence of social networks and the growing utilization of information technology has played a significant role in the emergence of extramarital relationships among young women. In this context, fostering sexual health self-care practices can serve as a preventive measure against the potential consequences of such relationships within this demographic.

This research unfolds in three stages:

1. Explanatory Sequential Mixed-Method Follow-up Study: Combining quantitative and qualitative phases.
2. Narrative Review: Involving a review of relevant literature.
3. Strategy Extraction through Expert Opinion: Finalizing strategies via a nominal group process.

Integrating quantitative and qualitative approaches, this study aims to comprehensively explore the younger generation's perspectives on psychological and social aspects of extramarital relationships, thereby providing robust documentation in this field.

## Background

Self-care involves the capacity of individuals, families, and communities to support their own health, prevent illnesses, maintain well-being, and manage health conditions, either with or without assistance from healthcare professionals [1]. Additionally, self-care has the potential to improve the use of preventive services, strengthen adherence to treatment, and reduce the need for healthcare services [2]. Research also indicates that self-care interventions can lead to cost savings within the healthcare system [3]. Self-care encompasses various domains, including self-awareness, self-testing, and self-management [4]. Self-awareness refers to the ability to monitor one's inner thoughts and feelings [5]. Self-testing involves the process of collecting samples, conducting tests, and privately interpreting the results to gain insights into one's health status [6]. Self-management is defined as an active or preventive approach aimed at sustaining a healthy lifestyle, preventing the onset of symptoms, assessing physical and mental changes, monitoring signs, and effectively coping with the impacts of diseases [7].

While self-care unquestionably provides numerous benefits and plays a crucial role in improving community health, it's important to acknowledge that in certain situations, self-care might unintentionally guide individuals toward using traditional or unconventional approaches to manage illnesses and seek treatment. This phenomenon frequently results from a combination of factors such as limited access to necessary healthcare resources, a lack of suitable medical services, and deeply rooted cultural beliefs and practices prevalent within particular communities [8].

The key distinction lies in the significant gap between self-medication and authentic self-care. Self-administering medication without a proper understanding of one's ailment does not align with the principles of self-care; instead, it has the potential to worsen the underlying illness [9].

Moreover, while self-care offers numerous advantages when it lacks support from governments and active involvement of health systems in organizing such care, it can place a financial burden on society. This can lead to increased out-of-pocket costs for individuals [10].

Adolescents are susceptible to participating in sexual activities, including high-risk sexual relationships, which can carry substantial threats to their reproductive and sexual well-being. The reproductive and sexual health of adolescents is impacted by cultural and social factors, and it hinges on their responsible decision-making and behavior, in line with the notion of self-care [11].

Self-care can be considered one of the primary and initial forms of women's healthcare. In today's world, as women become more aware, they actively seek ways to enhance their health, with self-care emerging as a key approach [12].

In a study conducted by Honarvar et al. to assess the attitudes and experiences of unmarried individuals regarding premarital sexual relationships, approximately 34% of the participants expressed their acceptance of such relationships. Shockingly, one out of every two unmarried individuals in the study reported engaging in premarital sexual activity, with nearly half of them having multiple sexual partners. The author highlights the alarming nature of the current situation, emphasizing the early age at which Iranian youth initiate sexual activity, the lack of awareness regarding sexually transmitted diseases, including HIV, and the existence of cultural and social barriers that hinder open discussions about sexuality [13].

Alimoradi et al. conducted a study with the aim of understanding the factors that shape the perceptions of Iranian adolescent girls regarding premarital sexual relationships. The study revealed that parents, teachers, and friends had the most significant influence on the sexual and fertility beliefs and actions of these adolescent girls. Additionally, the study highlighted the presence of peer pressure, which compelled adolescents to seek opposite-sex partners. Another noteworthy finding was the perspective of Iranian adolescents regarding protective societal norms. These norms included restrictions on sexual behaviors, the endorsement of sexual relations only within the bounds of marriage, the avoidance of premarital sex, and refraining from forming friendships with the opposite sex [14].

Engaging in sexual activity outside of marriage is viewed as illegal, irreligious, immoral, and socially taboo in Iran. Risky sexual behaviors, including unprotected sex, having multiple sexual partners, initiating sexual activity at a young age, can have detrimental effects on the health of young individuals. These behaviors may result in harmful health outcomes, as well as negative social and economic consequences [15].

In society, there is a noticeable contradiction and duality when it comes to premarital sexual relationships. While cultural and religious norms advocate for abstinence from such relationships, factors like widespread access to information technology, delayed marriage, and the prevalent use of virtual networks have led to a more discreet approach to sexuality among young people. As a result, this context provides an opportunity to explore and understand self-care practices among young women. The goal is to promote sexual health and, consequently, develop strategies for effective sexual health education programs.

The goal of this protocol study is to address the changing dynamics of sexual health among young, unmarried Iranian women in the context of shifting societal norms influenced by global changes. In response to the potential risks associated with extramarital sexual relationships, this research aims to develop a comprehensive set of self-care strategies to improve sexual health. This innovative study, conducted in three phases that include quantitative and qualitative research, a narrative review, and expert consultation, aims to gather evidence-based insights into the physical, psychological, and social changes affecting the sexual health of unmarried young women. Ultimately, its objective is to provide healthcare policymakers with essential knowledge for promoting the sexual well-being of this demographic.

## Methods/design

**Fig. 1:**
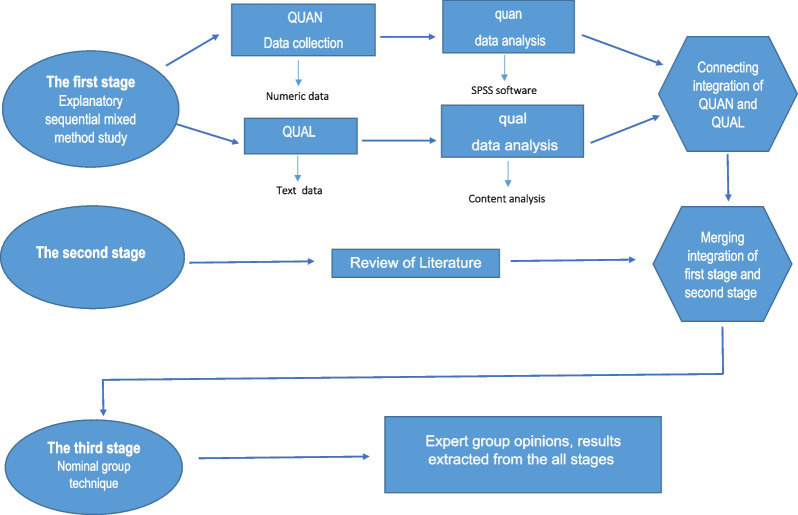
Study visual diagram

The visual diagram depicting the research procedure is presented in Fig. 1. Below are the details of the study.

### Aims

The primary aim of this study is to propose a protocol for developing self-care strategies to improve the sexual health of young, unmarried Iranian women. The overarching objectives of this study are as follows:

- To identify self-care practices and the factors associated with them in young women, with the goal of enhancing sexual health.
- To provide a comprehensive description of the self-care experiences of young, single Iranian women, as well as insights from other key informants, to promote sexual health.
- To determine effective self-care strategies for young, single Iranian women to enhance their sexual health.

### Study design

This research follows a three-stage approach:

- **Stage 1:** The initial stage consists of a sequential explanatory mixed-methods study, which includes both quantitative and qualitative phases. The quantitative phase will be conducted first, followed by the qualitative phase, aiming to provide a deeper understanding of respondents' experiences across the spectrum. Subsequently, the results from both phases will be integrated.
- **Stage 2:** The second stage involves a narrative review to examine existing strategies found in relevant literature.
- **Stage 3:** The third stage involves presenting the list of proposed strategies to a panel of experts. This panel will prioritize and extract the final self-care strategies for young women to enhance their sexual health.

#### Quantitative study

The quantitative phase of this study is a 6-month cross-sectional descriptive-analytical survey. This phase will concentrate on unmarried young female students who meet specific inclusion criteria and are enrolled at the Kerman University of Medical Sciences.

#### Sample size and sampling method

The minimum sample size needed to estimate self-awareness, self-testing, and self-management variables with a 95% confidence level and a precision level of 2.5, assuming a standard deviation of 25, was calculated using the following formula, resulting in a minimum sample size estimate of 400 individuals.

In this phase, data collection is conducted as follows:

- Kerman University of Medical Sciences offers education in various fields, encompassing 16 undergraduate programs, 36 master's degree programs, 3 general physician programs, 25 specialized doctoral programs, 19 clinical residency programs, and 5 sub-specialization programs.
- A random cluster is selected from each field using a random number table.
- Students who meet the study's inclusion criteria within the chosen cluster are offered the questionnaire, provided they express interest and consent to participate.
- After selecting the class, the researcher provides an introductory briefing to the students.
- Students access the questionnaire through a printed barcode, which they scan with their mobile phones. This allows them to answer the questions online.
- The informed consent form is located on the first page of the online questionnaire. After granting consent, students proceed to answer the questions.
- Additionally, the questionnaire includes an option for respondents to indicate "I do not want to answer this question."

### Inclusion criteria

The inclusion criteria for this study are as follows:

- Female gender
- Age between 18 to 29 years
- Willingness to provide informed consent
- Iranian nationality
- Meeting the definition of unmarried according to Iran's socio-cultural norms, which entails not having a permanent marriage record on one's birth certificate and registering marriages through official offices
- Enrollment as a student at any academic level, including undergraduate, master's, doctoral, specialty, and sub-specialty programs.

### Exclusion criteria

The sole exclusion criterion for this study is the student's lack of willingness to continue participating in the research.

### Scales and data collection

In the initial phase of the quantitative research, we will utilize a researcher-designed questionnaire to assess the perspectives of female university students regarding self-care and the factors influencing the maintenance of sexual health in young women. This questionnaire will be developed based on a review of relevant literature and existing tools, including studies by Bourdeau et al. [16], Barnes and Olson [17], Jaccard et al. [18], Najarkolaei et al. [19], Rahmani et al. [20, 21], Alimoradi et al. [11], Naghizadeh et al. [22], Simbar et al. [23], Khalajabadi Farahani [24], and the research team's practical experience. The questionnaire will consist of four main parts:

The questionnaire consists of four main parts:

- **Part 1:** This section gathers personal information about the study participants, including age, education, parents' education, ethnicity, economic status, internet and virtual network usage, communication with parents, discussions about sexual topics with parents and friends, and questions related to self-care.
- **Part 2:** This section focuses on the self-awareness of young female university students in achieving sexual health.
- **Part 3:** This section is dedicated to self-testing among young female university students as it pertains to achieving sexual health.
- **Part 4:** This section addresses the self-management practices of young female university students in achieving sexual health.

In the second, third, and fourth sections of the questionnaire, responses will be rated using a 5-point Likert scale, with the following scoring system: completely agree (5), agree (4), neutral (3), disagree (2), and completely disagree (1).

### Validity and reliability of the quantitative data collection tool

In this phase, a self-care questionnaire specifically designed to enhance sexual health among single young female students will be employed. To ensure the validity and reliability of this questionnaire, the following steps will be taken:

1. **Content validity assessment:** To assess content validity, the questionnaire will undergo both quantitative and qualitative evaluations by 10 relevant experts. They will use either the Content Validity Ratio (CVR) or Content Validity Index (CVI) methods. Additionally, 10 experts will assess its qualitative content validity, focusing on language, word usage, and item placement. Feedback from these experts will guide necessary revisions in consultation with the research team.
2. **Face validity assessment:** The questionnaire will be presented to 15 eligible female students to assess face validity. These students will provide feedback on its simplicity, comprehensibility, and clarity. Each question's importance will be rated using a 5-point Likert scale: highly important (score 5), moderately important (score 4), somewhat important (score 3), slightly important (score 2), and not important at all (score 1). An impact score will be calculated, with a criterion for quantitative face validity confirmation being an impact score exceeding 1.5.
3. **Reliability assessment:** To assess reliability, the test-retest method will be employed.

These steps will help ensure the questionnaire's validity and reliability for effective data collection.

### Data analysis

We will use descriptive statistics like frequency (percentage), mean, and standard deviation to depict demographic and socio-economic characteristics. Inferential statistics will serve these purposes:

- Independent samples t-test to assess relationships between quantitative variables and dichotomous qualitative ones like residence (city or village).
- Analysis of variance (ANOVA) to identify associations between quantitative variables and qualitative ones with multiple categories, such as education level.
- Pearson's correlation test to examine connections between two quantitative variables, like self-awareness and age.

### Qualitative study

In the qualitative phase of the study, content analysis will be employed to explore the experiences of single/unmarried young Iranian women and key informants concerning self-care for sexual health. This qualitative approach builds upon insights gathered from the quantitative phase and aims to provide a deeper understanding of self-care strategies for promoting sexual health in young women. Content analysis will help uncover and analyze the narratives and perspectives of the participants, enriching the overall findings of the study.

### Sampling method and data collection

In the qualitative phase of the study, participants will be selected to ensure maximum diversity in background factors. This includes variations in education, age, cultural and social status, religious affiliation, and economic status among the participants.

It's important to note that the research community for this project comprises students from the Kerman University of Medical Sciences. This university, categorized as a type 1 institution in Iran, enrolls several thousand students from various regions of the country. Therefore, the sample drawn from these students is not constrained by regional or provincial cultural specificities and can be considered representative of Iranian female students at large.

The diversity in participant selection will contribute to a comprehensive understanding of self-care strategies for promoting sexual health among young Iranian women. Data collection will involve in-depth interviews and other qualitative research methods to capture the rich experiences and perspectives of the participants.

### Sample selection and sample size

For this phase of the study, the selection of participating students will be carried out through targeted sampling, and the sampling process will continue until data saturation is achieved.

In qualitative research, there is no fixed or predetermined sample size. Instead, the determination of sample size is guided by the principle of data saturation. Data saturation is reached when gathering more data no longer leads to the emergence of new concepts or insights. In other words, it means that the researchers have thoroughly explored and understood the range of experiences and perspectives related to the research topic.

The sampling path in qualitative studies is flexible and adaptive, driven by the evolving understanding of the subject matter. If needed, data collection can persist beyond the point of saturation to ensure a comprehensive exploration of the research topic. This approach allows the research to capture a rich and diverse set of experiences and insights from the participants, ensuring that the qualitative phase of the study is thorough and informative [25].

The selection of female university students for the qualitative phase of the study was based on their active participation in the quantitative phase and their strong willingness to continue into the qualitative phase. These students were chosen because of the diversity in their responses regarding self-care practices for sexual health. They will be selected from Kerman University of Medical Sciences and will take part in semi-structured, in-depth interviews. This approach ensures that the qualitative phase captures a wide range of perspectives and experiences related to self-care for sexual health among young Iranian women.

The qualitative interview guide for female participants from Kerman University of Medical Sciences is designed to explore their perspectives on achieving sexual health through self-care. The semi-structured interviews will begin with open-ended questions and progress to inquiries about the participants' views on the significance of self-care in achieving sexual health. The interview guide includes the following questions:

1. When you hear the term “self-care for sexual health promotion,” what comes to mind?
2. What actions do you take to promote sexual health through self-care?
3. Are there any behaviors or practices you avoid to promote sexual health through self-care?
4. What factors or elements contribute to your success in promoting sexual health through self-care?
5. Conversely, what factors or elements hinder you from effectively promoting your sexual health through self-care?

These questions aim to elicit a comprehensive understanding of the participants' experiences, practices, and perspectives related to self-care for sexual health promotion.

In the qualitative phase of the study, all conversations with participants will be recorded with their explicit consent using a digital voice recorder. In addition to interviews with participants, key informants, such as fertility and sexual health experts specializing in sexual self-care for young individuals, will also be interviewed to enrich the study's findings.

The choice of interview locations will be based on participant preferences and circumstances to ensure their comfort and convenience. The duration of interviews will be adjusted according to the participant's willingness to engage in discussions and share their experiences during the process, allowing for flexibility in the interview process.

### Data analysis

Data analysis for the qualitative phase of the study will begin promptly once data collection commences. The analysis will follow the method proposed by Granheim and Luddmann. Here are the key aspects of the data analysis process:

- **Open coding:** The data will be analyzed through open coding. This involves systematically reviewing the interview transcripts and identifying categories based on recurring themes in response to the research questions. Open coding is a fundamental step in qualitative content analysis and helps in organizing and categorizing the data.
- **Qualitative content analysis:** The overall approach to data analysis is qualitative content analysis. This method allows for the extraction of meaningful insights and patterns from the qualitative data collected during the interviews. It involves a systematic examination of the data to identify themes, patterns, and categories that emerge from the participants' responses.

Regarding the integration of quantitative and qualitative data from the first phase, the results will be combined using the connection method. This integration will help provide a comprehensive understanding of self-care practices for sexual health among young Iranian women, drawing on both quantitative and qualitative perspectives.

### The second stage of the research: narrative review

The second stage of this study involves a narrative review with the aim of identifying self-care strategies relevant to enhancing sexual health among single/unmarried young women in existing literature. Here is an overview of the methodology:

- **Literature search:** Relevant texts will be sourced through keyword searches related to self-care and sexual health across various databases, including MEDLINE, Embase, CINAHL, Cochrane, Scopus, and Web of Science. Additionally, Persian databases such as Irandoc, SID, IranMedex, and Scimed will be queried using Persian keywords and their English equivalents.
- **Comprehensive search:** The objective of this stage is to gather insights, strategies, and effective approaches for promoting sexual health among young women on a global scale. To ensure comprehensive coverage, Google Scholar will also be consulted to identify and include relevant studies and texts in the review.

*Integration of the results of the first and second stages of the research:* Subsequently, the results obtained from the first and second stages of the research will be integrated through a side-by-side merging approach, and a list of the proposed strategies will be developed to be presented to the nominal group in the third stage.

### The third stage of the research: nominal group technique

In the third research stage, we'll use the nominal group technique to create effective self-care strategies for young women's sexual health. Here's an overview:

- Session with Experts: We'll gather sexual health and fertility experts and stakeholders.
- Sharing Strategies: We'll present proposed self-care strategies developed from previous research stages.
- Gathering Input: Participants will provide feedback to refine and prioritize the strategies.
- Compilation and Refinement: Strategies will be fine-tuned based on expert input, ensuring evidence-based effectiveness.

The nominal group technique leverages collective expertise for informed strategy development.

## Discussion

One of the fundamental challenges to the health of young people, especially single women, is engaging in risky sexual behaviors that can have negative consequences on their sexual health and fertility, such as unintended pregnancies, unsafe abortions, sexually transmitted infections, infertility, and more in their future lives [26, 27].

Usually, discussions around sex and gender-related topics create unpleasant feelings in individuals, thus little attention has been paid to these matters. Nevertheless, sexual needs are also human needs that should be addressed through necessary education and enjoyed. Unfortunately, it seems that within Iranian families, due to the lack of awareness and a lack of understanding of how to pass on accurate sexual information to adolescents and young people, sexual problems resulting from these issues are more prevalent [28]. A study conducted in 2012 on the fertility behaviors of female university students in Tehran showed that 50% of single female students had male friends, 23% had experienced some sort of sexual relations, and 10% had had sex. For all these reasons, research in the field of sexual issues, especially among adolescent and young age groups, has always faced challenges, and as a result, only a few studies have been conducted in this field in the country. However, even these few studies have indicated high-risk sexual behaviors among adolescents and young people in the country [29].

Sexual health is a field based on knowledge and skill development, so having scientific evidence is essential. Therefore, the results of this study can provide insights into the field of self-care for sexual health among single/unmarried young women and provide national documentation in the field of promoting strategies to relevant policymakers, planners, and program executives.

## Data Availability

Not applicable.
